# Harvesting Vibration Energy for Efficient Cocatalyst-Free Sonocatalytic H_2_ Production over Magnetically Separable Ultra-Low-Cost Fe_3_O_4_

**DOI:** 10.3390/ma17071463

**Published:** 2024-03-22

**Authors:** Kailai Zhang, Xiaodong Sun, Haijun Hu, Anqi Qin, Hongwei Huang, Yali Yao, Yusheng Zhang, Tianyi Ma

**Affiliations:** 1 Institute of Clean Energy Chemistry, Key Laboratory for Green Synthesis and Preparative Chemistry of Advanced Materials of Liaoning Province, College of Chemistry, Liaoning University, Shenyang 110036, China; zhangkl1996@163.com (K.Z.); s13940416997@163.com (H.H.); qanhdd@163.com (A.Q.); 2 School of Materials Science and Technology, China University of Geosciences, Beijing 100083, China; hhw@cugb.edu.cn; 3 Institute for the Development of Energy for African Sustainability (IDEAS), University of South Africa, Roodepoort 1710, South Africa; yaoy@unisa.ac.za; 4 School of Chemistry and Chemical Engineering, Hunan University of Science and Technology, Xiangtan 411201, China; yushengzhang@hnust.edu.cn; 5 School of Science, RMIT University, Melbourne, VIC 3000, Australia

**Keywords:** sonocatalysis, magnetically separable, Fe_3_O_4_, hydrogen production, charge separation

## Abstract

The cavitation effect is an important geochemical phenomenon, which generally exists under strong hydrodynamic conditions. Therefore, developing an economical and effective sonocatalyst becomes a vital method in capitalizing on the cavitation effect for energy generation. In this study, we first report a novel Fe_3_O_4_ sonocatalyst that can be easily separated using a magnetic field and does not require any additional cocatalysts for H_2_ production from H_2_O. When subjected to ultrasonic vibration, this catalyst achieves an impressive H_2_ production rate of up to 175 μmol/h/USD (where USD stands for dollars), surpassing most previously reported mechanical catalytic materials. Furthermore, the ease and efficiency of separating this catalyst using an external magnetic field, coupled with its effortless recovery, highlight its significant potential for practical applications. By addressing the key limitations of conventional sonocatalysts, our study not only demonstrates the feasibility of using Fe_3_O_4_ as a highly efficient sonocatalyst but also showcases the exciting possibility of using a new class of magnetically separable sonocatalysts to productively transform mechanical energy into chemical energy.

## 1. Introduction

Addressing the pressing demand for energy and confronting environmental concerns necessitate the transition from conventional fossil fuels to renewable and eco-friendly energy alternatives. Among these alternatives, hydrogen (H_2_) energy emerges as particularly promising, boasting advantages such as efficient combustion, abundant availability irrespective of fossil fuels, high energy content, lack of pollution, and superior efficiency. However, achieving environmentally friendly, user-friendly, and economically feasible hydrogen production remains a formidable challenge [1,2]. Photocatalytic materials have emerged as promising contenders for hydrogen generation through light-driven processes [3,4,5,6,7,8]. Despite their potential, challenges persist, notably in harnessing visible light effectively and optimizing electron–hole transfer rates, impeding their widespread adoption for large-scale hydrogen production. Similarly, electrocatalytic methods for hydrogen production, while efficient, face drawbacks such as energy consumption and the production of economically less viable by-products, hindering their extensive development [9,10,11,12,13]. However, a promising alternative in the realm of catalysis is mechanical catalysis. This innovative approach operates without the need for light or electrical stimulation, instead relying on mechanical stimuli to induce physical or chemical electron transfer, thereby facilitating catalytic processes. Since its inception, mechanical catalysis has garnered significant attention and found applications across various catalytic fields due to its distinct advantages and versatility. By leveraging mechanical forces, such as shear, compression, or vibration, mechanical catalysis offers a unique pathway to enhance reaction kinetics and selectivity, circumventing the limitations associated with traditional catalytic methods.

Furthermore, the versatility of mechanical catalysis extends beyond conventional catalytic reactions. It has been explored in diverse areas, including sustainable energy production, environmental remediation, and chemical synthesis, showcasing its potential to address complex challenges across multiple domains. Moreover, the simplicity and scalability of mechanical catalysis make it an attractive option for industrial applications, promising efficient and cost-effective solutions for hydrogen production and beyond. Piezocatalytic H_2_ production, as a subtype of mechanical catalysis, offers an intriguing avenue to substantially reduce catalysis costs by using mechanical energy, making it particularly attractive as it operates independently of light [14,15,16,17]. Piezoelectric effects are indeed restricted to crystals lacking a center of symmetry, limiting the range of semiconductor catalytic materials that can utilize mechanical energy for catalysis. However, an innovative approach in the form of sonoluminescence offers a promising alternative. Unlike piezoelectric and ferroelectric effects, sonoluminescence does not rely on material symmetry, allowing for broader application possibilities. This unique phenomenon involves the generation of light through the collapse of bubbles in a liquid medium, where appropriately positioned electrons and holes on the energy bands can initiate a cascade of reactions.

Recent research has witnessed a growing interest in leveraging non-piezoelectric semiconductor materials for effective sonocatalysis [18,19,20]. This trend indicates a shift towards exploring materials with diverse properties to overcome the limitations posed by crystal symmetry. Nevertheless, challenges persist in the preparation processes of these nanomaterials, which are often complex and yield unstable products. Additionally, the high rates of charge recombination associated with sonogenerated species present obstacles to the widespread application of these materials. Moreover, traditional heterogeneous sonocatalysts face issues regarding their separation from the reaction system post reaction, leading to potential losses and increased separation costs. This concern poses a significant barrier to the scalability and practicality of sonocatalyst development, hindering its transition from laboratory-scale research to real-world applications. Addressing these challenges requires a concerted effort to develop robust preparation methods for nanomaterials, improve their stability, and enhance their separation efficiency. Moreover, exploring novel catalytic architectures and materials with enhanced sonocatalytic properties could offer promising solutions to overcome existing limitations. By addressing these critical issues, the field of sonocatalysis can progress towards realizing its full potential as a viable and sustainable approach for various catalytic applications.

In light of the challenges associated with traditional sonocatalytic materials and their separation processes, the development of sonocatalytic materials tailored specifically for H_2_ production without the need for co-catalysts becomes imperative. These materials should possess key attributes such as cost-effectiveness, simplicity in synthesis, non-toxicity, minimal separation expenses, and exceptional performance. One promising approach involves the utilization of magnetic sonocatalytic materials, which offer several advantages over conventional counterparts. The application of an external magnetic field for assistance in material separation presents a non-destructive, straightforward, and user-friendly technique compared to other methods of liquid/solid separation. This approach not only simplifies the separation process but also reduces costs and minimizes potential losses associated with traditional separation methods. Ferro-ferric oxide (Fe_3_O_4_), commonly known as magnetite, exhibits a distinctive inverse spinel structure and occurs naturally, making it an attractive candidate for magnetic sonocatalytic materials. Notably, the synthesis cost of Fe_3_O_4_ is lower compared to many previously reported sonocatalytic materials, enhancing its appeal for large-scale applications [21]. Furthermore, Fe_3_O_4_ has demonstrated wide-ranging applications in sonocatalytic degradation reactions, showcasing its potential as a versatile catalyst material. Despite its promising attributes and extensive applications in degradation reactions, surprisingly, there have been no reports on H_2_ production through sonocatalysis using magnetic Fe_3_O_4_ material. This presents an exciting opportunity for further exploration and research in the field of sonocatalysis, particularly in the context of hydrogen generation. By leveraging the unique properties of Fe_3_O_4_ and exploring its catalytic potential in H_2_ production, researchers can potentially unlock a novel and efficient pathway for sustainable hydrogen generation.

In this study, we first present the groundbreaking use of magnetic Fe_3_O_4_ material in sonocatalytic H_2_ production reaction. When exposed to ultrasonic vibration, this catalyst achieves a remarkable H_2_ production rate of up to 175 μmol/h/USD. Additionally, this catalyst can be quickly separated using an external magnetic field and easily retrieved, showcasing its considerable promise for real-world applications. This pioneering work opens up new horizons for the application of magnetic Fe_3_O_4_ in the field of sonocatalytic H_2_ production, offering potential benefits in terms of cost-effectiveness and environmental sustainability. One of the key advantages of this catalyst is its ease of separation using an external magnetic field. This feature allows for quick and efficient retrieval of the catalyst from the reaction mixture, highlighting its practical utility for real-world applications. Moreover, the simplicity of the separation process contributes to the overall cost-effectiveness and feasibility of utilizing magnetic Fe_3_O_4_ in sonocatalytic H_2_ production. Furthermore, our pioneering work not only showcases the impressive performance of magnetic Fe_3_O_4_ as a sonocatalyst but also opens up new avenues for its application in the field of hydrogen production. By leveraging the unique properties of Fe_3_O_4_ and exploring its catalytic potential, we have laid the foundation for further advancements in sustainable H_2_ production technologies.

## 2. Materials and Methods

### 2.1. Materials

All chemicals were obtained from commercial sources and used without further purification. Commercial magnetic Fe_3_O_4_ powder was purchased from Tianjin Damao Chemical Reagent Co., Ltd. (Tianjin, China). Basic information of commercial magnetic Fe_3_O_4_ powder is displayed in Table 1. The deionized water was used throughout the experiment.

### 2.2. Sample Characterization

The crystalline structure of the sample was analyzed using X-ray powder diffraction (XRD) with Cu Kα radiation (λ = 0.15418 nm) via a Bruker D8 instrument (Karlsruhe, Germany). Field emission scanning electron microscopy (SEM) from ZEISS (Oberkochen, Germany), and transmission electron microscopy (TEM) from JEM-2100F (Tokyo, Japan), were employed for visualizing the morphology and structure of the sample. The surface state and element composite of the samples were examined by X-ray photoelectron spectroscopy (XPS) using an XPS spectrometer (ESCALAB 250Xi, Thermo Fisher Scientific, Waltham, MA, USA) with an Al Kα X-ray source. Fourier transform infrared spectroscopy (FTIR) data were collected using a NICOLET IS10 spectrophotometer from Thermo Fisher Scientific (Waltham, MA, USA). Magnetic properties were characterized using a LakeShore7404 Vibrational Sample Magnetometer (VSM) instrument (Westerville, OH, USA). Thermogravimetric analysis (TGA) was carried out in a nitrogen environment with a METTLER TOLEDO TGA/SDTA851 instrument (Columbus, OH, USA).

### 2.3. Electrochemical Tests

Electrochemical impedance spectroscopy (EIS), Nyquist plots, and sonocurrent measurements were conducted on an electrochemical workstation (CHI 760E, Shanghai, China). The electrolyte used was a 0.2 M Na_2_SO_4_ solution, with Pt serving as the counter electrode and Ag/AgCl as the reference electrode. To prepare the working electrode, 10 mg of the synthesized samples was uniformly dispersed in 1 mL of ethanol via ultrasound treatment. Subsequently, this suspension was evenly coated onto indium tin oxide (ITO) glass. The ultrasound frequency and ultrasound power used in the electrochemical tests were fixed at 50 kHz and 240 W.

### 2.4. Experimental Procedure

The sonocatalytic H_2_ production performance of sonocatalysts was evaluated in an ultrasonic cleaner (Kunshan) with an adjustable power range of 120, 180, and 240 W and an adjustable frequency range from 50 to 75 kHz. The temperature of the reaction system was controlled at 25 °C (with a variation of ±2 °C) through the circulation of water and an ice bag to minimize the influence of temperature fluctuations. Typically, in a sealed Pyrex reactor with a total volume of 500 mL, 0.05 g of Fe_3_O_4_ powder was added to a mixture of 180 mL deionized (DI) water and 20 mL methanol (MeOH) or Triethanolamine (TEOA) or glycol (EG). Subsequently, the reaction system was completely evacuated to eliminate any residual air, and internal and external pressures were equalized using high-purity (99.99%) N_2_ for approximately 60 min. Following this, the suspension underwent ultrasonic treatment for sonocatalytic H_2_ production reaction. To quantify the produced amount of H_2_, 1 mL of gas was collected from the reaction system at 30 min intervals and analyzed using a gas chromatograph (GC-9790, Taizhou, China).

## 3. Results

### 3.1. Material Systhesis and Characterization

**Figure 1:**
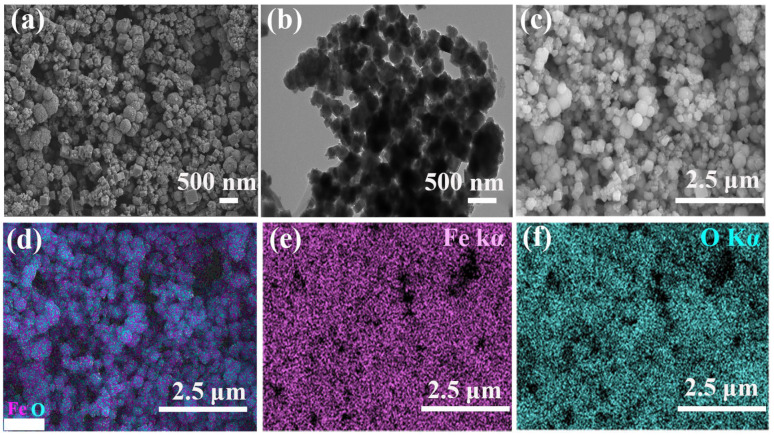
(**a**) SEM image of magnetic Fe_3_O_4_; (**b**) TEM image of magnetic Fe_3_O_4_; (**c**–**f**) SEM and EDS elemental elemental mapping (Fe Kα and O Kα) of magnetic Fe_3_O_4_.

The investigation into the morphology and microstructure of the sample was conducted using a combination of advanced microscopy techniques, including scanning electron microscopy (SEM) and transmission electron microscopy (TEM). The results, showcased in Figure 1a,b, provide detailed insights into the particle-like morphology of magnetic ferro-ferric oxide (Fe_3_O_4_). In addition, we also utilized particle size analysis software ImageJ V1.8.0 for a comprehensive evaluation of the sample’s particle size distribution, which showed that the particle size distribution range was 50~400 μm, and the average particle size distribution range was about 206 ± 9 nm (Figure S1). The particle-like morphology of Fe_3_O_4_ plays a crucial role in facilitating good diffusivity within the sonocatalytic system. This morphology promotes the efficient diffusion of reactant molecules to the active sites on the catalyst surface. By enhancing the accessibility of reactants to these active sites, the Fe_3_O_4_ particles effectively contribute to accelerating the catalytic reaction rates. Further analysis through energy-dispersive spectrometer (EDS) element mapping images, as depicted in Figure 1c–f, not only confirmed the presence of Fe and O within the magnetic Fe_3_O_4_ structure but also unveiled the uniform distribution of these elements throughout the material. The overall EDS distribution chart, representing the elemental composition of magnetic Fe_3_O_4_, can be observed in Figure S2, further clearly indicating the presence of two elements: Fe and O. Notably, a large peak located at about 1.7 keV was observed, which is an intrinsic attribute of the silicon substrate in the measurement of SEM-EDS mappings. The total spectral distribution diagram, as illustrated in Table 2, presents the total spectrum of the synthesized material. An evident observation from the data is the striking similarity between the mass fraction ratios of Fe and O obtained through the EDS test and the theoretical mass fraction values of Fe and O. This close alignment between the experimental and theoretical values provides compelling evidence affirming the successful synthesis of the material.

**Figure 2:**
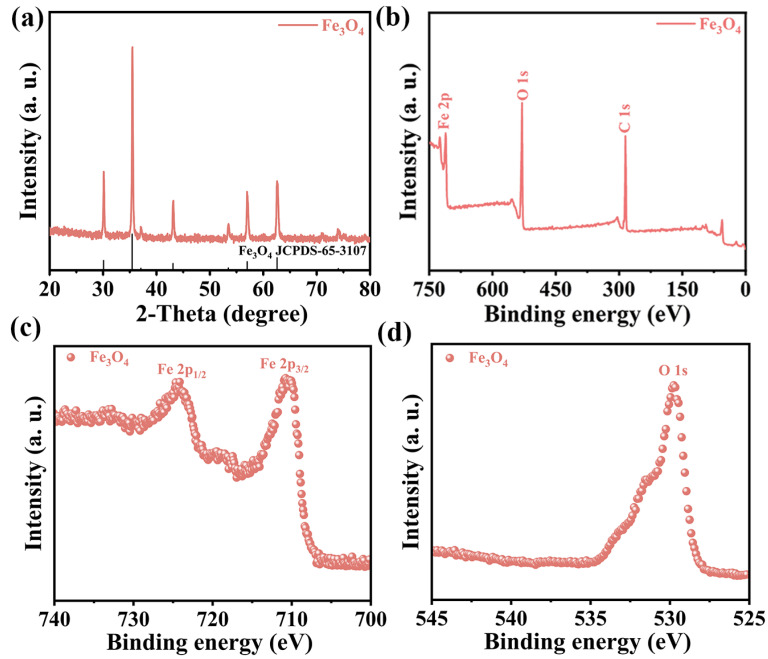
(**a**) XRD of magnetic Fe_3_O; (**b**) XPS survey spectrum of magnetic Fe_3_O_4_; (**c**) high-resolution Fe 2p of magnetic Fe_3_O_4_; (**d**) high-resolution O 1s of magnetic Fe_3_O_4_.

The X-ray diffraction (XRD) analysis conducted at room temperature provided crucial insights into the crystal structure and phase composition of the catalytic material. Figure 2a displays the XRD patterns of magnetic Fe_3_O_4_, where the diffraction peaks at 2θ = 30.46°, 35.35°, 43.68°, 54.31°, 57.13°, and 63.75° corresponded to the (220), (311), (400), (422), (511), and (440) crystal planes of Fe_3_O_4_, consistent with JCPDS 65-3107. Furthermore, no additional peaks were detected, confirming the absence of crystalline phases of FeO and Fe_2_O_3_ and indicating the high purity of the sample. To investigate the chemical composition and atomic chemical environment of the catalyst, X-ray photoelectron spectroscopy (XPS) analysis was performed. The binding energy of all elements was corrected with reference to C 1s. The XPS survey spectra revealed the presence of O, Fe, and C elements in magnetic Fe_3_O_4_, with element C attributed to instrument contamination (Figure 2b). Furthermore, high-resolution XPS spectra of Fe 2p are presented in Figure 2c, where the peak at 710.8 eV corresponded to Fe 2p_3/2_, while the peak at 724.2 eV corresponded to Fe 2p_1/2_. This indicated the existence of Fe elements in Fe_3_O_4_, consistent with previous findings in the literature [22]. The high-resolution XPS spectrum of O 1s also verified the existence of O (Figure 2d).

**Figure 3:**
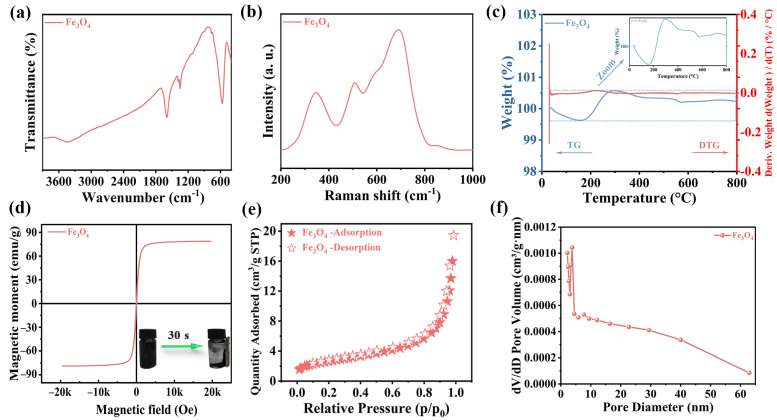
(**a**) FT-IR spectra of magnetic Fe_3_O_4_; (**b**) Raman spectrum of magnetic Fe_3_O_4_; (**c**) TG and DTG of magnetic Fe_3_O_4_; (**d**) VSM of magnetic Fe_3_O_4;_ (**e**) N_2_ adsorption/desorption of magnetic Fe_3_O_4_; (**f**) pore size distribution of magnetic Fe_3_O_4_.

Moreover, Figure 3a presents the Fourier Transform Infrared Spectroscopy (FT-IR) spectra of magnetic Fe_3_O_4_ nanoparticles. The FT-IR spectra revealed a prominent stretching absorption peak at 567 cm^−1^, which corresponded to the Fe-O-Fe group of magnetic Fe_3_O_4_. Additionally, peaks around 1635 and 3420 cm^−1^ correspond to hydroxyl groups associated with water, aligning with previously reported FT-IR data for magnetic Fe_3_O_4_ [23]. The Raman spectrum of magnetic Fe_3_O_4_ is shown in Figure 3b, and its typical peak position is consistent with the previously reported literature [24]. In addition, thermogravimetric (TG) and differential thermal gravimetry (DTG) analysis of these nanoparticles (Figure 3c) showed negligible mass loss throughout the heating process. Additionally, Vibrating Sample Magnetometer (VSM) measurements were used for characterizing the magnetic properties of materials. VSM measurements under a ±15 kOe magnetic field (Figure 3d) revealed a saturation magnetization (Ms) of approximately 78.80 emu/g. The hysteresis loop of Fe_3_O_4_, passing through the origin without hysteresis, residual magnetization, or coercivity, confirmed the superparamagnetic nature of the material, facilitating easy separation from solutions via an external magnetic field. N_2_ adsorption/desorption measurements for Brunauer–Emmett–Teller (BET) surface area analysis (Figure 3e) indicated a specific surface area (S_BET_) of 9.60 m^2^/g for magnetic Fe_3_O_4_. This relatively low S_BET_ suggested that the sonocatalytic hydrogen production primarily occurs on the surface of the magnetic Fe_3_O_4_, rather than within the material’s internal structure. Through further analysis of the data, it can be seen that the pore size distribution is about 2.4 nm, which belongs to the mesoporous range, and the pore volume is about 0.03 cm^3^/g (Figure 3f).

### 3.2. Sonocatalytic H_2_ Production

**Figure 4:**
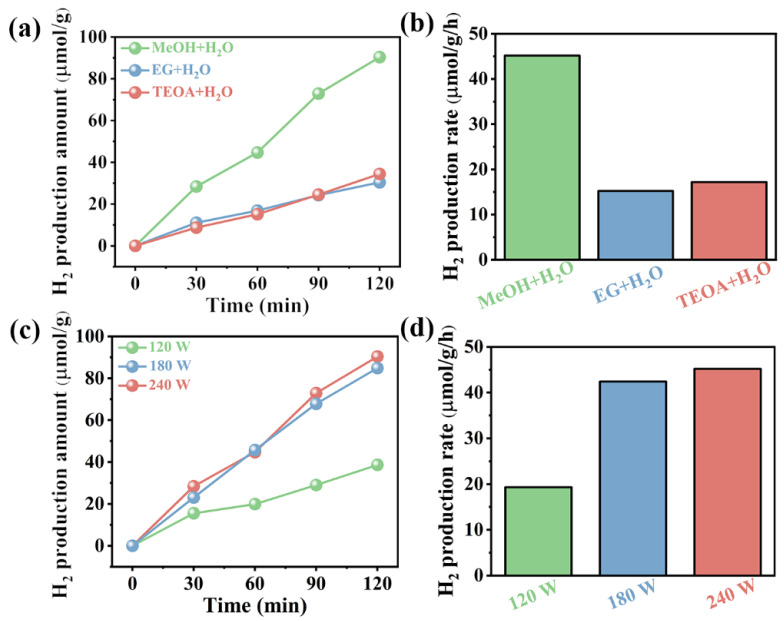
(**a**) Time profiles of sonocatalytic H_2_ production amount by Fe_3_O_4_ with different sacrificial agents (reaction conditions are as follows: ultrasonic frequences = 50 kHz; ultrasonic powers = 240 W; and sacrificial agent = MeOH, EG, or TEOA); (**b**) H_2_ production rates of Fe_3_O_4_ under ultrasound with different sacrificial agents (reaction conditions are as follows: ultrasonic frequences = 50 kHz; ultrasonic powers = 240 W; and sacrificial agent = MeOH, EG, or TEOA); (**c**) time profiles of sonocatalytic H_2_ production amount by Fe_3_O_4_ with different powers (reaction conditions are as follows: ultrasonic frequences = 50 kHz; ultrasonic powers = 240, 180, or 120 W; and sacrificial agent = MeOH); (**d**) H_2_ production rates of Fe_3_O_4_ with different powers (reaction conditions are as follows: ultrasonic frequences = 50 kHz; ultrasonic powers = 240, 180, or 120 W; and sacrificial agent = MeOH).

The sonocatalytic H_2_ production performance of magnetic Fe_3_O_4_ was assessed under ultrasonic vibration, without any co-catalyst deposition, using various sacrificial agents. Notably, compared with other sacrificial agents, when using methanol (MeOH) as a sacrificial agent, the magnetic Fe_3_O_4_ showcased superior H_2_ production performance at 45.1 μmol/g/h (Figure 4a,b), which may be because MeOH can capture the holes most effectively. Prior research indicated that power is a critical factor in sonocatalysis. Consequently, the impact of varying ultrasonic powers on H_2_ production performance by magnetic Fe_3_O_4_ was investigated (Figure 4c,d). The results showed an increased trend in sonocatalytic performance with increasing power. This can be attributed to the fact that higher vibrational energy leads to increased stress, which in turn generates more sonoelectric charges on the catalyst’s surface, consequently enhancing the hydrogen production rate [25]. In addition, the impact of varying ultrasonic frequences on H_2_ production performance by magnetic Fe_3_O_4_ was investigated (Figure S3). The results showed an increased trend in sonocatalytic performance with increasing frequences. This improvement could be ascribed to the enhanced mass transfer phenomenon achieved by increasing the ultrasonic frequency [26]. To eliminate the influence of inherent hydrogen generation capabilities of methanol/H_2_O, control experiments without any variables were carried out (Figure S4). These experiments revealed that, in the absence of a sonocatalyst, a minimal hydrogen production rate of 0.44 μmol/h was observed in a methanol/water mixture subjected to ultrasonic vibration. Nonetheless, the introduction of Fe_3_O_4_ into this methanol/water system resulted in an increase in hydrogen production, elevating the rate from 0.44 μmol/h to 2.25 μmol/h.

**Figure 5:**
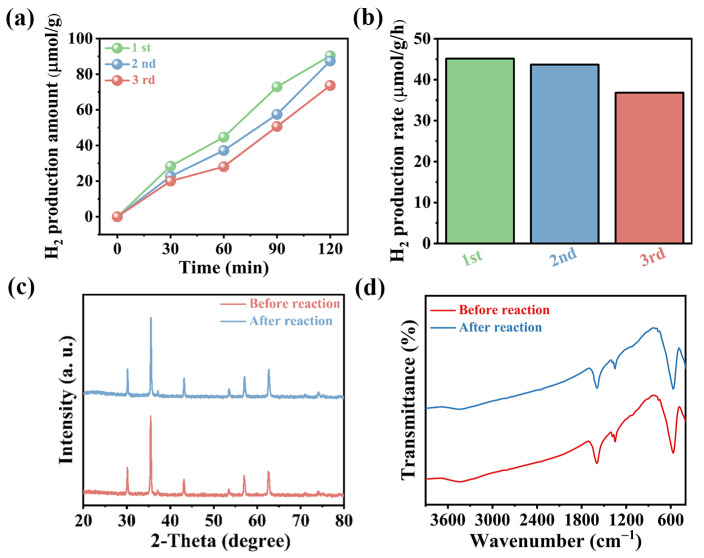
(**a**) Time profiles of sonocatalytic H_2_ production by Fe_3_O_4_ under 3 cycles (reaction conditions are as follows: ultrasonic frequences = 50 kHz, ultrasonic powers = 240 W, and sacrificial agent = MeOH); (**b**) H_2_ production rates of Fe_3_O_4_ after 3 cycles (reaction conditions are as follows: ultrasonic frequences = 50 kHz, ultrasonic powers = 240 W, and sacrificial agent = MeOH); (**c**) the XRD spectra over magnetic Fe_3_O_4_ before and after sonocatalytic water splitting reactions; (**d**) the FT-IR spectra over magnetic Fe_3_O_4_ before and after sonocatalytic water splitting reactions.

In addition, the cyclic stability of magnetic Fe_3_O_4_ was also examined (Figure 5a,b), revealing a minor decrease in H_2_ production performance over three successive cycles, likely due to the gradual loss of the Fe_3_O_4_ catalyst. The reusability and stability of magnetic Fe_3_O_4_ in sonocatalytic hydrogen generation were further confirmed by a series of characterization analysis before and after the sonocatalytic reaction. Figure 5c shows the PXRD pattern of the sample before and after undergoing a sonocatalytic reaction. While the range of diffraction angles remained largely unchanged, there was a noticeable reduction in peak intensity. This showed that the crystal form of magnetic Fe_3_O_4_ had not changed after the sonocatalytic reaction, but the crystal structure had become imperfect. In addition, Figure 4d shows the FT-IR spectrum of the sample before and after the sonocatalytic reaction. The peak position has basically not changed, but the intensity has decreased, which also verified the high stability of the catalytic material. All these characteristics proved that the sonocatalytic materials have excellent cyclic stability. In fact, Fe_3_O_4_ demonstrates versatility as it can serve not only as a sonocatalyst but also as a thermal and triboelectric catalyst. To ascertain that the hydrogen production attributed to ultrasound is a result of the material’s sonoelectric effect rather than frictional or thermal effects, a comprehensive analysis was conducted along with a controlled trial. While Fe_3_O_4_ could theoretically serve as a thermal catalyst, our experiments were carefully designed with stringent temperature control. A cooling water system was utilized to maintain constant temperatures, minimizing any thermal effects. Based on this, the hydrogen production observed in our study is not a result of thermal influences. Moreover, to address the possibility of friction-induced hydrogen production, a specific experiment was conducted by focusing on triboelectric catalysis. This involved examining hydrogen production of Fe_3_O_4_ in a methanol aqueous solution under room temperature stirring conditions, with the findings presented in Figure S5. The observed hydrogen production was negligible, reinforcing our stance that the hydrogen generation in our experiments is not attributable to triboelectric effects. In addition, when evaluating semiconductor catalytic technologies, considering economic factors is crucial. Currently, existing semiconductor catalytic technologies, while claiming to achieve efficient water splitting for hydrogen production, often entail high catalyst synthesis costs. Although these reported hydrogen-producing catalysts appear to perform exceptionally well based on simple hydrogen production performance data, the actual production costs in practical applications often far exceed the economic benefits they bring. This is an issue that deserves attention. Therefore, assessing the hydrogen production performance of semiconductor catalysts in terms of hydrogen yield per unit cost (μmol/h/USD) is more reasonable compared to the commonly used hydrogen yield per gram per hour (μmol/g/h). This approach better reflects the economic feasibility of semiconductor catalysts as it directly relates their performance to production costs, aiding in determining the competitiveness of specific semiconductor catalysts in real-world applications. As a result, conducting a comprehensive cost–benefit analysis regarding the application potential of magnetic Fe_3_O_4_ becomes imperative. This study showcases a remarkable H_2_ production rate of 175 μmol/h/USD, surpassing the performance of numerous previously reported mechanical catalytic materials, as delineated in Table 3 [27,28,29,30,31,32]. This finding underscores the promising prospects of magnetic Fe_3_O_4_ as a highly efficient and cost-effective sonocatalyst for hydrogen production.

### 3.3. Sonocatalytic Mechanism Investigation

**Figure 6:**
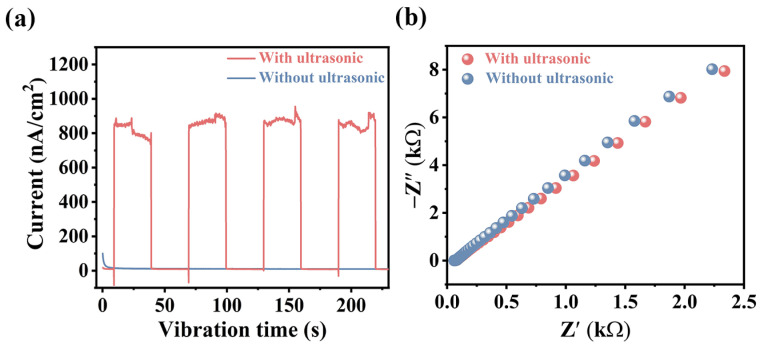
(**a**) Current obtained from magnetic Fe_3_O_4_ under ultrasonic or without ultrasonic vibration; (**b**) EIS plots obtained from magnetic Fe_3_O_4_ under ultrasonic or without ultrasonic vibration.

The separation and transfer efficiency of sonocarriers was studied through a series of electrochemical measurements [33]. As shown in Figure 6a, the impact of ultrasonic vibration on the system was vividly depicted, showcasing a remarkable increase in the average current density, reaching approximately 920 nA post introduction of ultrasonic vibration. This surge in current density signifies the effectiveness of ultrasound in facilitating the separation of sonocarriers within the Fe_3_O_4_ medium, consequently driving the sonocatalytic H_2_ production reaction. Moreover, upon cessation of the ultrasound, the sonocurrent swiftly plummeted to 0, further underscoring the direct correlation between ultrasound-induced vibrations and the dynamic behavior of sonocarriers. Additionally, the arc radius of the electrochemical impedance spectrum (EIS) curve of magnetic Fe_3_O_4_ under ultrasonic vibration was slightly smaller than that of magnetic Fe_3_O_4_ without ultrasonic vibration (Figure 6b). These results both indicated that mechanical energy introduction into the catalyst system effectively reduced charge transfer impedance and enhanced the separation and transfer efficiency of charges.

**Figure 7:**
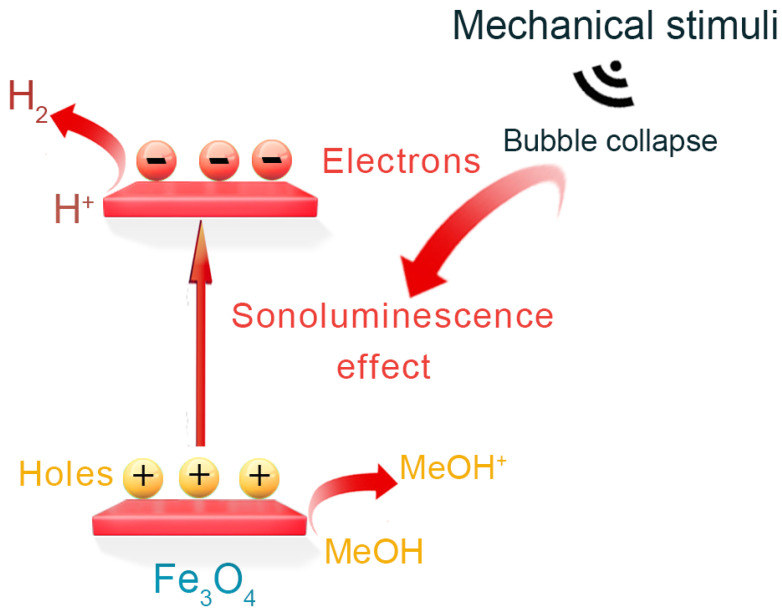
The illustration of sonocatalytic H_2_ production process over Fe_3_O_4_.

The mechanism of H_2_ production from magnetic Fe_3_O_4_ by sonocatalysis, as depicted in Figure 7, elucidates the intricate interplay of various physical and chemical processes underlying this phenomenon. This process begins with the sonocatalytic water splitting, facilitated by the Fe_3_O_4_ catalyst, where H_2_O acts as the reactant and MeOH is used as the sacrificial agent. Under the influence of ultrasonic vibrations, a pivotal driving force attributed to the phenomenon of ultrasonic cavitation, the Fe_3_O_4_ catalyst is activated. Ultrasonic cavitation involves the rapid oscillation, expansion, and subsequent collapse of small bubbles in a liquid medium when exposed to ultrasonic waves, leading to the generation of localized regions of high temperature and pressure [18,34,35,36,37,38,39,40,41]. This environment, accentuated by the phenomenon of sonoluminescence—the emission of light and energy from the collapsing bubbles—energizes electrons to the conduction band of Fe_3_O_4_, leaving behind holes in the valence band. This electron excitation primes the catalyst for activity, enabling the reduction of hydrogen ions (H^+^) to form H_2_, while oxygen atoms produced from water splitting typically react with sacrificial agents, producing oxidation products. The sacrificial agent MeOH is crucial in this process. It is oxidized during the reaction, providing more reactive electrons to the system by consuming the holes created during water splitting and preventing their recombination with electrons, thereby ensuring continuous H_2_ production. This oxidation process typically results in the formation of formaldehyde, formic acid, and other oxidation products [42,43,44,45,46,47,48,49]. Overall, the intricate interplay of sonoluminescence-induced activation, electron excitation, and sacrificial agent utilization collectively contribute to the efficient sonocatalytic production of hydrogen, highlighting the pivotal role of ultrasonic vibrations in driving catalytic reactions and unlocking the potential of Fe_3_O_4_-based materials for sustainable energy applications.

## 4. Conclusions

In summary, our study has demonstrated the exceptional potential of magnetically responsive Fe_3_O_4_ nanoparticles in facilitating H_2_ production via sonocatalysis, achieving an impressive rate of 175 μmol/h/USD. This accomplishment highlights the efficacy of harnessing vibrational energy, a key aspect of sonocatalysis, to drive catalytic reactions efficiently. Moreover, the unique property of Fe_3_O_4_ nanoparticles to be rapidly and efficiently separated and recovered using an external magnetic field adds another layer of practicality to their utilization in real-world applications. This capability not only simplifies the catalyst recovery process but also minimizes material loss and enhances the overall sustainability of the process. Furthermore, the robust durability, economic viability, widespread accessibility, environmental compatibility, and remarkable stability exhibited by Fe_3_O_4_-based materials underscore their significance as highly promising candidates for future applications in energy conversion processes. These materials hold immense potential in converting mechanical energy into chemical energy, particularly in the context of sustainable energy technologies.

## Figures and Tables

**Table 1:** Basic information of commercial magnetic Fe_3_O_4_ powder.

Sample	Appearance	BET (m^2^/g)	Size (nm)
Fe_3_O_4_	Black powder	≈10	50–400

**Table 2:** Total spectral distribution diagram.

Element	Line Type	wt%	wt% Sigma	at%
O	k-line	26	0.16	55
Fe	k-line	74	0.16	45
Total amount:		100		100

**Table 3:** The H_2_ production rate for the preparation cost of different mechanical catalytic materials.

Sample	Cost	H_2_ Production Rate (μmol/h/USD)	Ref.
Fe_3_O_4_	0.036 ¥/g	175	This work
Ti_3_C_2_TX	5.58 ¥/g	34	[27]
MoS_2_	137.44 ¥/g	0.028	[28]
Bi_2_WO_6_	46.48 ¥/g	0.58	[29]
BaTiO_3_	9.25 ¥/g	1.41	[30]
BiFeO_3_	30.99 ¥/g	0.57	[31]
Pd-BiFeO_3_	180.12 ¥/g	0.90	[32]

## Data Availability

The data presented in this study are available on request from the corresponding author (due to privacy).

## Supplementary Materials

The following supporting information can be downloaded at https://www.mdpi.com/article/10.3390/ma17071463/s1. Figure S1: The size distribution bar chart of Fe_3_O_4_; Figure S2: The EDS distribution chart of the total number spectrum of Fe_3_O_4_; Figure S3: The sonocatalytic H_2_ production rates of catalyst under different frequencies; Figure S4: The sonocatalytic H_2_ production blank experiment; Figure S5: The tribocatalysis H_2_ production performance of Fe_3_O_4_; Figure S6: Reaction system used for sonocatalytic H_2_ production driven by ultrasonication; Figure S7: Detection system used for sonocatalytic H_2_ production driven by ultrasonication.
