# Supplementary material for: Harvesting Vibration Energy for Efficient Cocatalyst-Free Sonocatalytic H2 Production over Magnetically Separable Ultra-Low-Cost Fe3O4
Source: Materials (Basel). 2024 Mar 22;17(7):1463. doi: 10.3390/ma17071463 (PMC11012934; doi:10.3390/ma17071463)
Supplement: Supplementary file 1 [file materials-17-01463-s001.zip › materials-2921690-supplementary.pdf]

Supporting Information

Harvesting Vibration Energy for Efficient Cocatalyst-Free Sonocatalytic H_2 Production over Magnetically Separable Fe_3O_4

Kailai Zhang[1], Xiaodong Sun[1,*], Haijun Hu[1], Anqi Qin[1], Hongwei Huang[2], Yali Yao[3], Yusheng Zhang[4] and Tianyi Ma[5,*]

[1] Institute of Clean Energy Chemistry, Key Laboratory for Green Synthesis and Preparative Chemistry of Advanced Materials of Liaoning Province, College of Chemistry, Liaoning University, Shenyang 110036, China

[2] School of Materials Science and Technology, China University of Geosciences, Beijing, 100083, China

[3] Institute for the Development of Energy for African Sustainability (IDEAS), University of South Africa, Roodepoort 1710, South Africa

[4] School of Chemistry and Chemical Engineering; Hunan University of Science and Technology, Xiangtan 411201, China

[5] School of Science, RMIT University, Melbourne, VIC 3000, Australia

* Correspondence: sunxiaodong@lnu.edu.cn (X. S.); tianyi.ma@rmit.edu.au (T. M.)

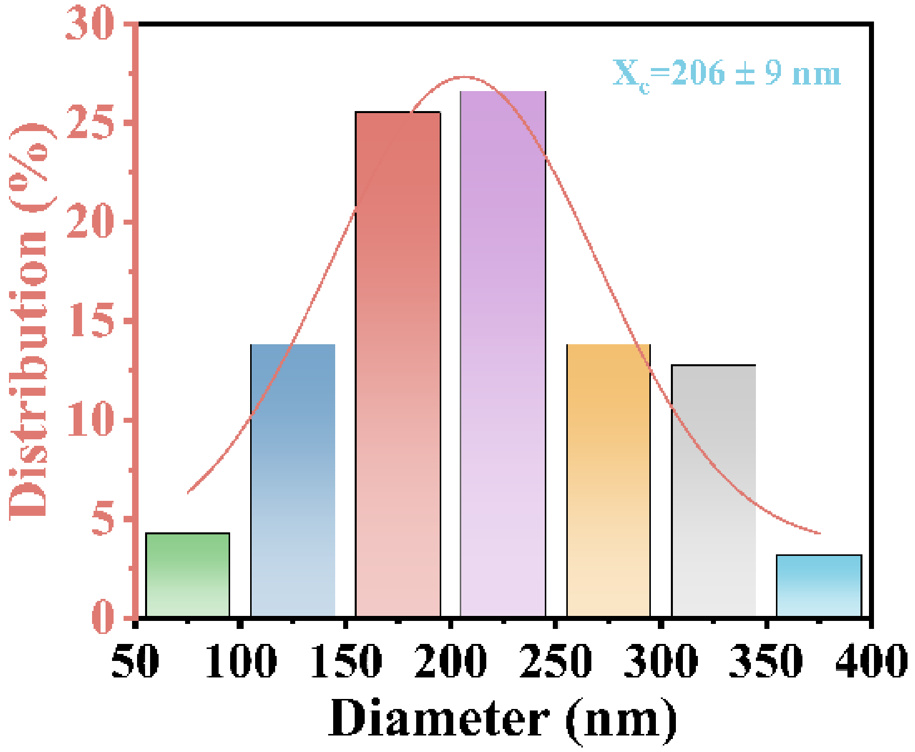

Figure S1. The size distribution bar chart of Fe_3O_4.

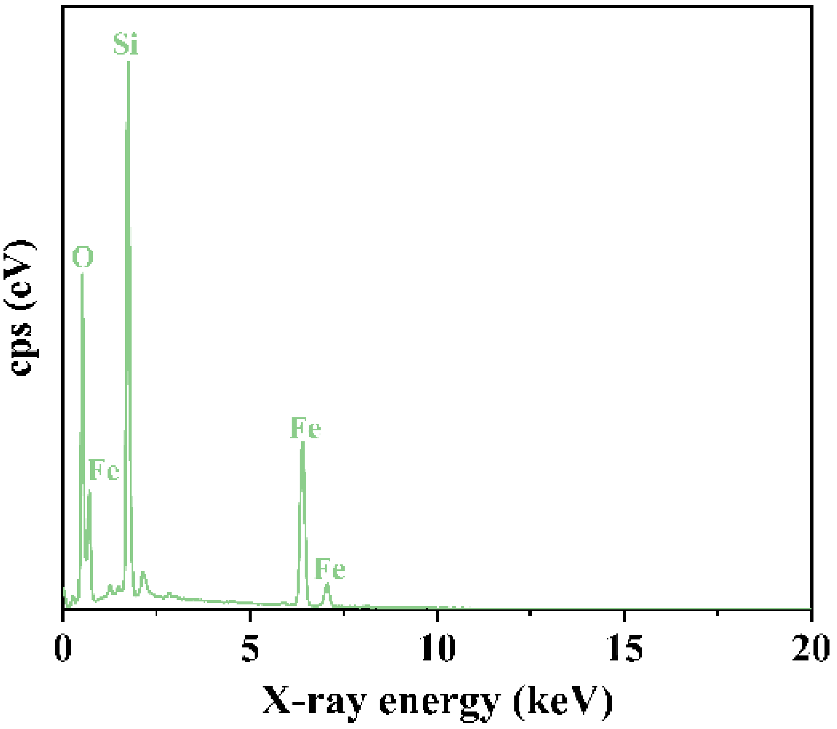

Figure S2. The EDS distribution chart total number spectrum of Fe_3O_4.

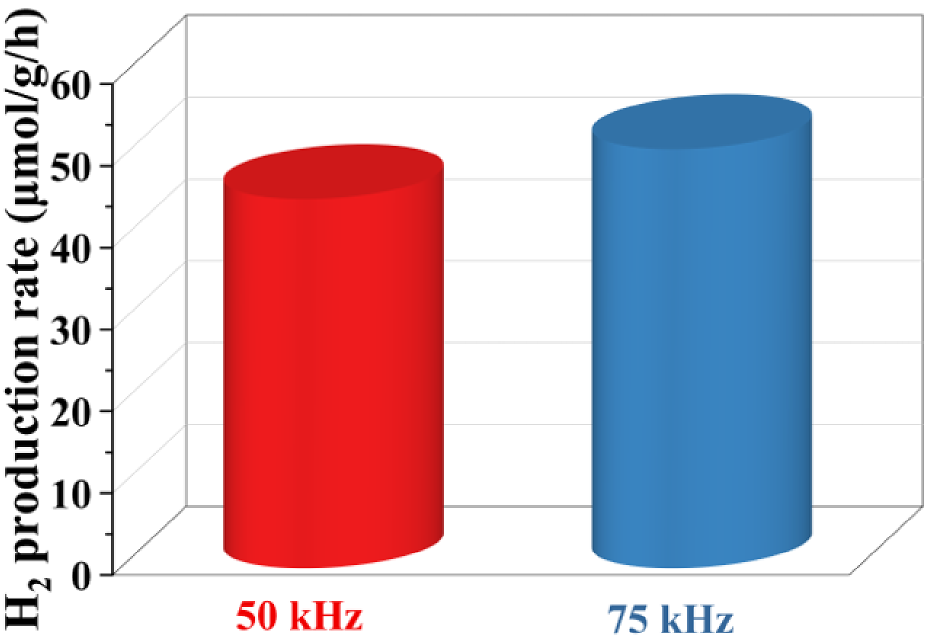

Figure S3. The sonocatalytic H_2 production rates under the condition of 240 W with different frequencies (50 or 75 kHz) of $MeOH+H_2O+Fe_3O_4$ (reaction conditions are as follows: ultrasonic frequences = 50 or 75 kHz, ultrasonic powers = 240 W and sacrificial agent = MeOH). In addition, the impact of varying ultrasonic frequences on H_2 production performance by magnetic Fe_3O_4 was investigated. The results showed an increased trend in sonocatalytic performance with increasing frequences. The improvement could be ascribed to the enhanced mass transfer phenomenon achieved by increasing the ultrasonic frequency.

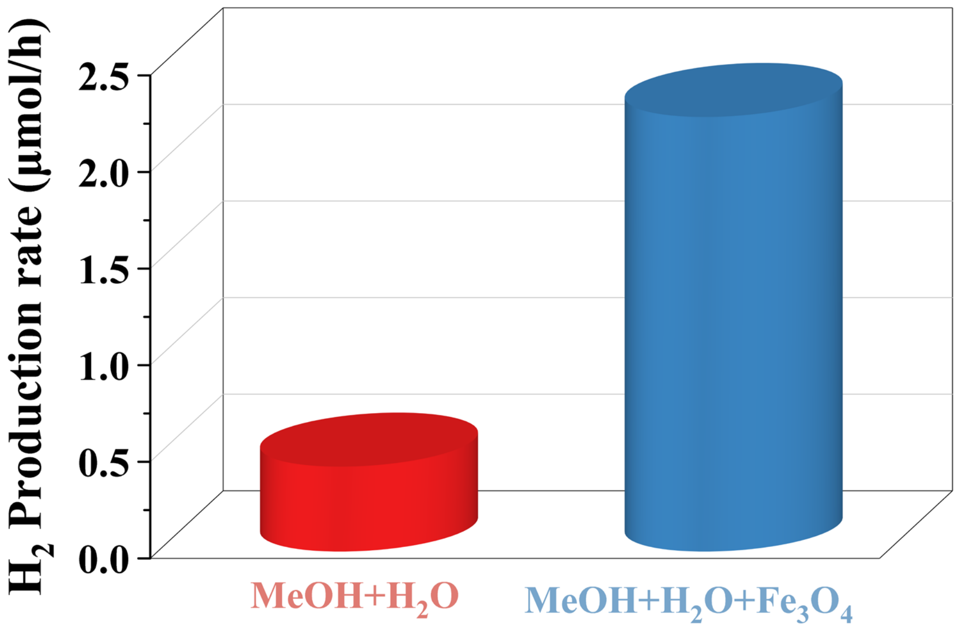

Figure S4. The sonocatalytic H_2 production rates under the condition of only $MeOH+H_2O$ and $MeOH+H_2O+Fe_3O_4$ (reaction conditions are as follows: ultrasonic frequences = 50 Hz, ultrasonic powers = 240 W and sacrificial agent = MeOH). To exclude the effect of MeOH self-producing H_2 performance, corresponding blank controls experiments were conducted. It is shown that in the absence of a sonocatalyst, a low H_2 yield of 0.44 μmol/h was generated in an $MeOH/H_2O$ solution under ultrasonic vibration. However, when the Fe_3O_4 was added into the above $MeOH/H_2O$ system, the H_2 production rate increased from 0.44 to 2.25 μmol/h.

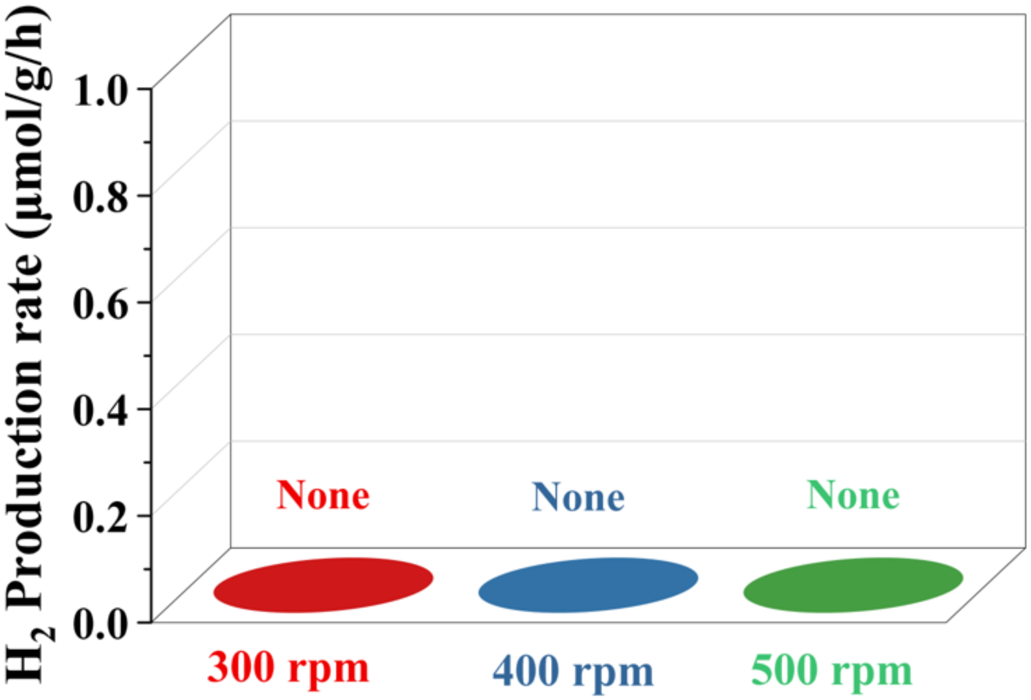

Figure S5. The Tribocatalysis H_2 production performance of $MeOH$+H_2O+Fe_3O_4 at different stirring rates. To address the possibility of friction-induced hydrogen production, we conducted a specific experiment focusing on triboelectric catalysis. This involved examining hydrogen production of Fe_3O_4 in a methanol aqueous solution under room temperature stirring conditions. The observed hydrogen production was negligible, reinforcing our stance that the hydrogen generation in our experiments is not attributable to triboelectric effects.

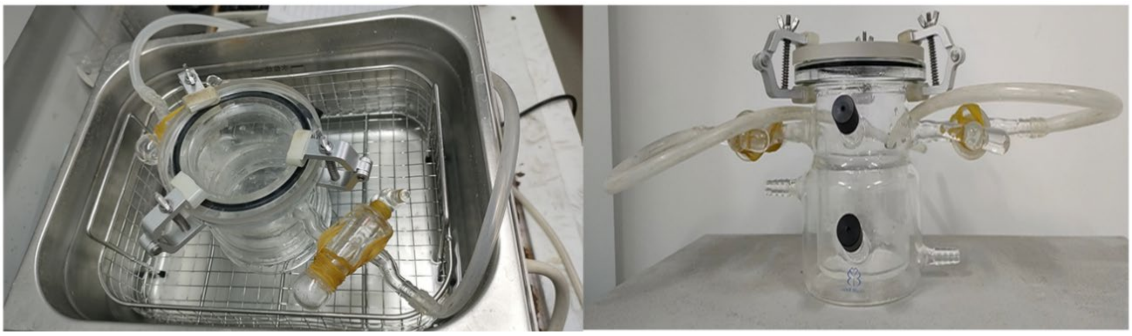

Figure S6. Reaction system used for sonocatalytic H_2 production driven by ultrasonication.

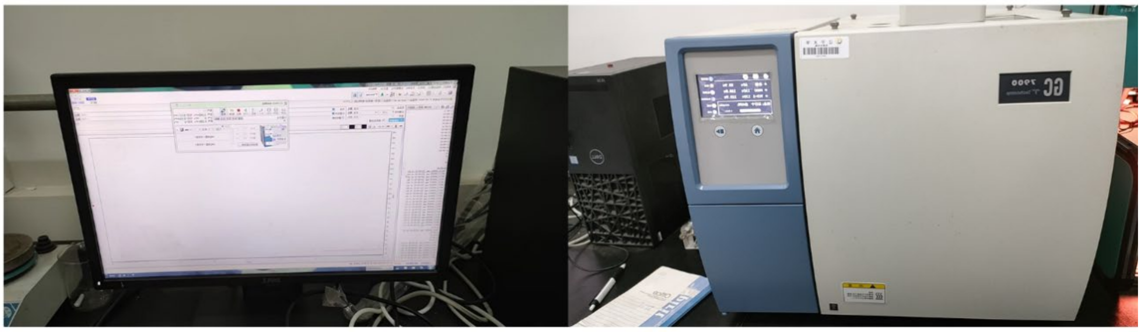

Figure S7. Detection system used for sonocatalytic H_2 production driven by ultrasonication.
